# Emerging Piezo1 signaling in inflammation and atherosclerosis; a potential therapeutic target

**DOI:** 10.7150/ijbs.63819

**Published:** 2022-01-01

**Authors:** Shafiu A. Umar Shinge, Daifang Zhang, Ahmad Ud Din, FengXu Yu, YongMei Nie

**Affiliations:** 1 Cardiovascular Surgery Department, Affiliated Hospital of Southwest Medical University, Luzhou, Sichuan PRC.; 2 Clinical Research Center, Southwest Medical University, Luzhou, Sichuan PRC.; 3 Drug Discovery Research Center, Southwest Medical University, Luzhou, Sichuan PRC.; 4 Collaborative Innovation Center for Prevention and Treatment of Cardiovascular Disease of Sichuan Province, Southwest Medical University, Luzhou, Sichuan PRC.

**Keywords:** Atherosclerosis, Piezo1 channel, Endothelial cell, Inflammation, shear stress, mechanomedicine

## Abstract

**Purpose of Review:** Atherosclerosis is the principal cause of cardiovascular diseases (CVDs) which are the major cause of death worldwide. Mechanical force plays an essential role in cardiovascular health and disease. To bring the awareness of mechanosensitive Piezo1 role in atherosclerosis and its therapeutic potentials we review recent literature to highlight its involvement in various mechanisms of the disease.

**Recent Findings:** Recent studies reported Piezo1 channel as a sensor, and transducer of various mechanical forces into biochemical signals, which affect various cellular activities such as proliferation, migration, apoptosis and vascular remodeling including immune/inflammatory mechanisms fundamental phenomenon in atherogenesis.

**Summary:** Numerous evidences suggest Piezo1 as a player in different mechanisms of cell biology, including immune/inflammatory and other cellular mechanisms correlated with atherosclerosis. This review discusses mechanistic insight about this matter and highlights the drugability and therapeutic potentials consistent with emerging functions Piezo1 in various mechanisms of atherosclerosis. Based on the recent works, we suggest Piezo1 as potential therapeutic target and a valid candidate for future research. Therefore, a deeper exploration of Piezo1 biology and translation towards the clinic will be a novel strategy for treating atherosclerosis and other CVDs.

## 1. Introduction

Atherosclerosis is a chronic inflammatory disease 1-4, resulting from several factors 5. It is the leading cause of fatality and morbidity globally 6, 7. Atherosclerosis is the multifactorial disease associated with various mechanisms such as inflammation, immune, and other cellular processes related to hyperlipidemia, obesity, diabetes, and associated metabolic disorders. Atherosclerosis prevalence is gradually increasing due to global increase in obesity and diabetes, leading to increase in cardiovascular diseases (CVDs) burden. As estimated, global death from CVDs may go beyond 23.6 million by the year 2030 8. The importance of mechanical forces in cardiovascular physiology and pathology have been reported for years. Endothelial cells (EC) sense mechanical force in the form of shear stress and transduce the force into biological signals through various membrane proteins 9, 10 including Piezo1. Various mechanical cues sensed by Piezo1 affect multiple signaling pathways, which lead to alterations in functions, phenotype, gene expressions and cell behavior including proliferation, migration, apoptosis and remodeling, the crucial features of atherosclerosis. It is increasingly clear from multiple clinical evidences and *in vivo* experimental subjects 9, 11 that mechanical force in form of low and oscillatory flow at the branching and curved vessel regions generates atherogenic shear, leading to EC inflammatory responses thereby developing distinct phenotype to distinctively interact with traditional risk factors and facilitate for the preferential site for vulnerability of atherosclerosis 12, 13. In recent years, an outstanding multidisciplinary effort in research has led to an exciting discovery of novel mechanosensitive ion channels Piezo 1 & 2, which bring us to a new era of mechano-transduction research. The channels are specialized with versatile functions of various mechanosensing, including stretching, membrane tension, cyclic pressure, shear stress and other mechanical cues. In addition to their identified functions, increasing evidence shows that their roles are not restricted to cardiovascular but in various aspects of cellular biology, including endothelial endocrine and paracrine aspects 14, 15. Different abnormalities or up/downregulation of cellular activities were observed in several studies following the mutation, silencing/blocking, deletion, cell specific or global knockout and deficiency of Piezo1 14, 16-22 based on its specific function. Due to Piezo1 physiological significance, its gain or loss-of-functioning mutants was found to correlate with various diseases in humans, including hereditary stomatocytosis 23 and generalized lymphatic dysplasia 24-26 indicating Piezo1 functional significance and its therapeutic potentials.

## 2. Piezo discovery

Mechanotransduction is the translation of mechanical forces into biochemical signals, which is necessary for potent cellular physiology. The molecular identity of mammalian mechanosensitive channels remained poorly understood, until the evolutionary discovery of Piezo channel family, which shed light on this mystery 27, 28. This opens the door for more concepts on the distinctive molecular identities of these channels and mechanotransduction mechanisms for sensory signaling. Patapoutian team revealed in 2010 that Piezo1 and Piezo2 proteins, encoded by the Piezo1/FAM38A and Piezo2/FAM38B genes, and recognized as mechanically activated (MA) cation channels, which are necessary in mammalian cell physiology. Piezo proteins are evolutionary conserved cellular mechanosensors that are necessary for proper multicellular physiology 29. They were first recognized as potential channels in the neuroblastoma cell line (Neuro2A) through electrophysiology, combining whole cell stimulation/recording patch clamp, and molecular biology. Multiple cell types were mechanically stimulated by Coste et al. seeking for a cell line with a robust and repeatable mechano-responsiveness and he discovered it in Neuro2A. the authors examined approximately 75 genes and found one named Fam38A as important. The protein has about 2500 amino acids long and exhibits no similarity to other channels and was called Piezo1. Cloning of a homologues gene (named Piezo2) from cell of dorsal root ganglion (DRG) and searching of the databases indicated that there were homologues genes in numerous animals as well as plants 29, 30. Piezo2 currents were comparable to Piezo1 but it had significantly differential kinetics as well as conductance; with faster inactivation and a low unitary conductance and low level of expressions. Using quantitative PCR, the researcher found that RNA coding for Piezos concentrations differs in bladder, lung and skin with extremely high amounts of Piezo1. Whereas significant expression of Piezo2 was found in DRG neurons 31. When piezo proteins are exposed to mechanical force, including shear stress, swelling, tension and compression, they release cations, which then promote cellular excitations and transmission of signals. Piezo1 is engaged in a variety of essential biological processes, including vascular 32 and lymphatic valve development 33, 34, blood pressure regulation, RBC volume regulation, bladder endothelial cell and renal tubular epithelial cell force sensing, and bone formation 14, 29. In mammals, Piezo2 mediates the mechanotransduction process in somatosensation of pain, touch and proprioception 35. Piezo1 and Piezo2 are substantially expressed in several mechanosensitive tissues in mouse. For instance, Piezo1 is abundant in mechanosensitive cells in the, bladder, skin, lung, kidney, and colon, while Piezo2 exhibits high expression in dorsal root ganglia primary sensory neurons, and in the lung, colon and bladder 29, 36. The relevance of EC Piezo1 in vasculature biology was initially identified in recent study, and constitutive Piezo1 knockout (KO) was embryonically fatal in few days due to obvious deformity in developing vessels in mouse 37, 38. However, Piezo2 was demonstrated necessary for mechanotransduction in Merkel cells, Piezo2 disrupting in the skin, exhibits decreased static firing ratio in mouse and reduced behavioral response to gentle touch stimuli 39. Thus, authentically proving that Piezos serves physiological function for *in vivo* mechanosensing. Following their discovery, a number of studies were conducted on Piezo proteins architecture, their, cellular distribution in mammals, physiological functions and associated pathologies, and molecular mechanisms were demonstrated 40, 41 as discussed in this review. Convincingly, these findings proved that Piezo 1 and Piezo2 are genealogical products correlated with cellular mechanosensation.

## 3. Piezo structure

Piezo is the family of genes, Piezo1 and Piezo2, and are large transmembrane proteins that represent a special class of mechanically activated (MA) cation channels. Human (MA) channel molecular identity was mysterious for long, until the revolutionary discovery of Piezo 1& 2, which may increase our understanding of cell signaling and mechano-transduction, bringing new opportunities for understanding and targeting theses signaling mechanisms involved in health and disease. Piezo1 protein identical to that of humans was initially discovered in a mouse. Piezo1 proteins are collectively arranged to form a three blade-like propeller, it is inserted within the lipid bilayer which makes the central ion pore that senses mechanical forces 42-45. Piezo1 extracellular propeller domain serves as a sensor of flow related-shear and other mechanical stress 46, 47. As observed on the cryo-electron microscope, several features of the channel morphology of mouse Piezo1, which is identical to that of humans, were identified. In respect to structure and function, Zhao et al. classified Piezo1 into three functional segments; (1) the ion-conduction pore portion which is C-terminal (2) the anchor; CTD, and the beam, which are the transducing elements**,** and (3) the TM blade-constituting mechanosensory portion 48, the structure of the channel are also deeply reviewed in the following pieces of literature 43, 45, 49. It was earlier disclosed by various structural and functional studies that Piezo1 channel plays a versatile functions and demonstrated its association with other membrane proteins such as PECAM (platelet endothelial cell adhesion molecule) 14 ECM (extracellular matrix) FAZ (focal adhesion zones) AJs (adherence junctions) and cytoskeletal structures 27 etc., the Piezo1 interplay with cytoskeleton in mechano-sensing was comprehensively reviewed in detail by Nourse et al 27, 50*,* and for broad structural concept in recent paper 49. Currently almost all of our understanding of the structure-function correlation of Piezo channels came from Piezo1 studies, while Piezo2 studies are so limited. However, the structure of full-length 2,822-residues of mice Piezo2 homo-trimers was determined to a resolution of 3.6-3.8, disclosing a totally resolved topography of 38 transmembrane helixes as well as a completely closed pore containing both transmembranous and cytosolic constriction regions. The enhanced Piezo2 specimen, which was solubilized in the detergent glycol-diosgenin (GDN), enabled the first acquiring of an overall 3.8-resolution cryo-electron microscope (cryo-EM) architecture that resembled the Piezo1 three-bladed, propeller-shaped architecture. All of the structural subunits seen in Piezo1 were resolved to 3.0 to 3.5 resolutions in the structure of Piezo2, with the exceptions of the extracellular cap (which was almost unresolved). In comparison to Piezo2, the Piezo1 cap domain rotates clockwise 51. The outer part of the inner and outer helices, and the top transmembrane gates of Piezo1, display a clear outward extension in a clockwise manner related to Piezo2, but the adjacent TM25-TM36 display a more modest and anticlockwise motions. When only the inner helix and the subsequent C-terminal subunit were overlaid, similar alterations in the inner helix and upper transmembrane gate were detected. Piezo1's outer part of the inner helix shows an outward extension of approximately a helical width (about 5.3) in comparison to Piezo2 51, 52. These alterations cause the top gate radius to increase from 0.9 for L2743 in Piezo2 to 4.5 for L2469 in Piezo1. Furthermore, when compared to Piezo2, the cap, beam subunits, and distal blade of the three Piezo1 structures are clearly displaced. Remarkably, the transmembrane gate - but not the cytosolic constriction neck - is dilated in all three Piezo1 structures, demonstrating that the differences in transmembrane pore conformation between the Piezo1 and Piezo2 structures are unlikely to be driven by detergent differences. Indeed, among the three Piezo1 structures, dilatation of the top transmembrane gate L2469 is strongly linked to cap domain displacement 49, 51.

### 3.1 Piezo channels structure-function relationship

As widely expressed in various cell types including cardiomyocytes, EC, VSMCs, and immune cells, Piezo1 play a wide range of roles in cellular physiology as well as pathology. Regarding this, researchers were curious in exploring the structures and/or domain of the channel involves in its function in health and disease. Despite revelation of general structure of Piezo1 by cryo-electron microscope (cryo-EM), the exact structural regions/subunits involved in activating as well as consequent inactivating of Piezo1 in correlation with its function are to be clarified. Through electrophysiology various researches study the possible structural domains to explore Piezo1 activation 53 and Piezo1&2 inactivation kinetics amid mechanical stimulation 54. Beside several structural studies 44, 55, Lewis et al revealed three tiny structural subunits in the extracellular cap, which could independently confer the distinctive kinetics of Piezo2 inactivating over Piezo1. Through cysteine crosslink the author demonstrated that conformational flexibilities of these subunits are required for mechanical activating to happen and that electrostatic interacting functionally copulate the cap to the extensive blades that was suggested to serve as sensors of membrane curvature as well as tensions. Both Piezo1 and Piezo2 mutating was found in patients diagnosed with distal arthrogryposis and xerocytosis 16, 56 that induces a gain-of-function mutants through delaying the inactivating, demonstrating that normal functions require perfect inactivation of the channel involving these subunits 54. On the other hand, activation Piezo1 activation was demonstrated to involves concerted movements of the, outer helix, inner helix, anchor subunit, CTD, latch and beam subunits. Recent studies demonstrated that E2133 mutating influence single channel conducting as well as ion selectiveness, and sensitivity to the pore blocking agent ruthenium red 57. In human *Piezo1*, the similar mutating of R2482H leads to delayed inactivating kinetics and is correlated with dehydrated hereditary stomatocytosis 23, 58, 59. These data imply that the anchor subunit-inner helixes interacting in controlling the Piezo1 opening as well pore characteristics. Interestingly, human subject with dehydrated hereditary stomatocytosis have the similar mutants in R1353P, which could probably disrupt or kink the helical beam at this location and lead to slowing inactivating kinetics. Hence, the distal beam ending might be a structural determinant of gating features, potentially via interacting with the CTD 59. These structures of Piezo1 present the high resolution image of structural and functional relationship of this unique ion channel. The common human disorder of *Piezo1* and *Piezo2* gain of function mutating could be mapped into the structures, demonstrating that these models cover multiple of the functionally essential subunits and, furthermore, provides for a structural interpreting of the disease causative processes.

### 3.2 Piezo1 activation and inactivation mechanisms

Previous studies demonstrated Piezo1 activation mechanisms as in the concept of “Force-from-lipids” 60, 61, showing the force sensing mechanism of Piezo1 via the bilayer and unequivocal cytoskeletal engagement, including ECM. But the precise activation mechanism and how Piezo1 sense and transduce mechanical forces including the molecular and conformational alterations during the process is yet unknown. Using the advantages of recent advancement of structural and functional studies using cryo-electron microscope 51, 59, patch clamp electrophysiology 62, 63 and atomic force microscope 64 has brought invaluable awareness to the structure, functions and conformational alterations of Piezo1 channel in relation to its activation mechanisms. In these studies, various structures that contains the Piezo1 core site (Table 2) was lately disclosed 43, 63 and suggested their importance in the channel activation mechanisms by different mechanical, and chemical stimuli 50 (Table 3), as well as voltage gating 65, in association with other proteins, such as extracellular matrix (ECM) 66 and cytoskeletal structure 27, including two proposed mechanisms referred as “force-from-lipids” 45, 67 and “force-from-filaments”^50^. Shear stress considerably elevate membrane tension through augmenting membrane fluidity that encourages the phospholipid molecule activity, results in larger membrane tension 68, and membrane tension was reported to triggered Piezo1 with great intensity at 1.4 mN/m 69, 70. Recent study disclosed the possible mechanism and structural alteration which occurs upon mechanical gating of Piezo1, the study reported that in the absence of tensions, the curvaceous form of Piezo1 is balanced in an innate-analogue model membrane, producing indented membrane with trilobal topography. While Piezo1 flattens and expands its propeller domain for adopting stretched bilayer amid stretch application, leading to alteration in the beam helix tilting angle. Those activities lead to tilt and lateral motion of the pore lining of TM37 and TM38 helices, result in the gating of the channel and moving of lipid which occupies outer area of Piezo1 pore domain. The author further revealed that the Piezo1 structural flattening recognized by their work discloses C-terminal extracellular domain (CED) likely by shear stress, which was enclosed in the membrane before channel opening 71, demonstrating the mechanosensing and gating mechanism of Piezo1 channel including shear detection. Furthermore, Shi et al demonstrated the inhibition of PIEZO1 channel inactivation by sphingomyelinase (SMPD3) through accelerating the generation of ceramides (an important SMPD3 product), which favors the activating state of the channel over closed state like inactivating states. The author revealed that neutral sphingomyelinase antagonists as well as sphingomyelin phosphodiesterase 3 (SMPD3) genealogical interruption induces Piezo1 transition into a highly inactivating state. He further shows that ceramide also restores non-inactivating channel functions. Sphingomyelin (substrates of SMPD3), unlike ceramide, does not influence inactivation but does influence channel force sensitivities. Sphingomyelin (substrates of SMPD3), unlike ceramide, does not influence inactivation but does influence channel force sensitivities. This indicated sphingomyelinase activities, ceramide, and sphingomyelin as regulators of native Piezo gating, which permits prolong activities 72. In addition to these outstanding findings, Scheuring and colleagues demonstrated that Piezo1 is a springy structure that normally bends the cellular membrane where it lies, but will flatten out when for instance a mechanical force is applied to the cell membrane. “As the membrane tension increases, the structure of Piezo1 flattens and stretches out to occupy a larger space, which in turn gates the ion channel”, as stated by Scheuring. He also realized that other stimulus stretches and flattens Piezo1 structure, like that of a pulling force on its arms from internal or on outer domain (CED) from outer side of the cell, in principle can gate the channel, making it an appropriately adoptable mechanism for various cell types and physiological roles to which it operates 73. Scheuring et al. also reported in Nature that, cryo-electron microscope showed that, in lipid vesicles of various sizes Piezo1 adapts distinct degrees of curvature 64. Altogether these results demonstrate a great achievement and step forward toward elucidating the precise gating mechanism of Piezo1 and its integral structure for internal gating motions involved in Piezo1 mechanotransduction.

## 4. Piezo1 functions

**Figure 1:**
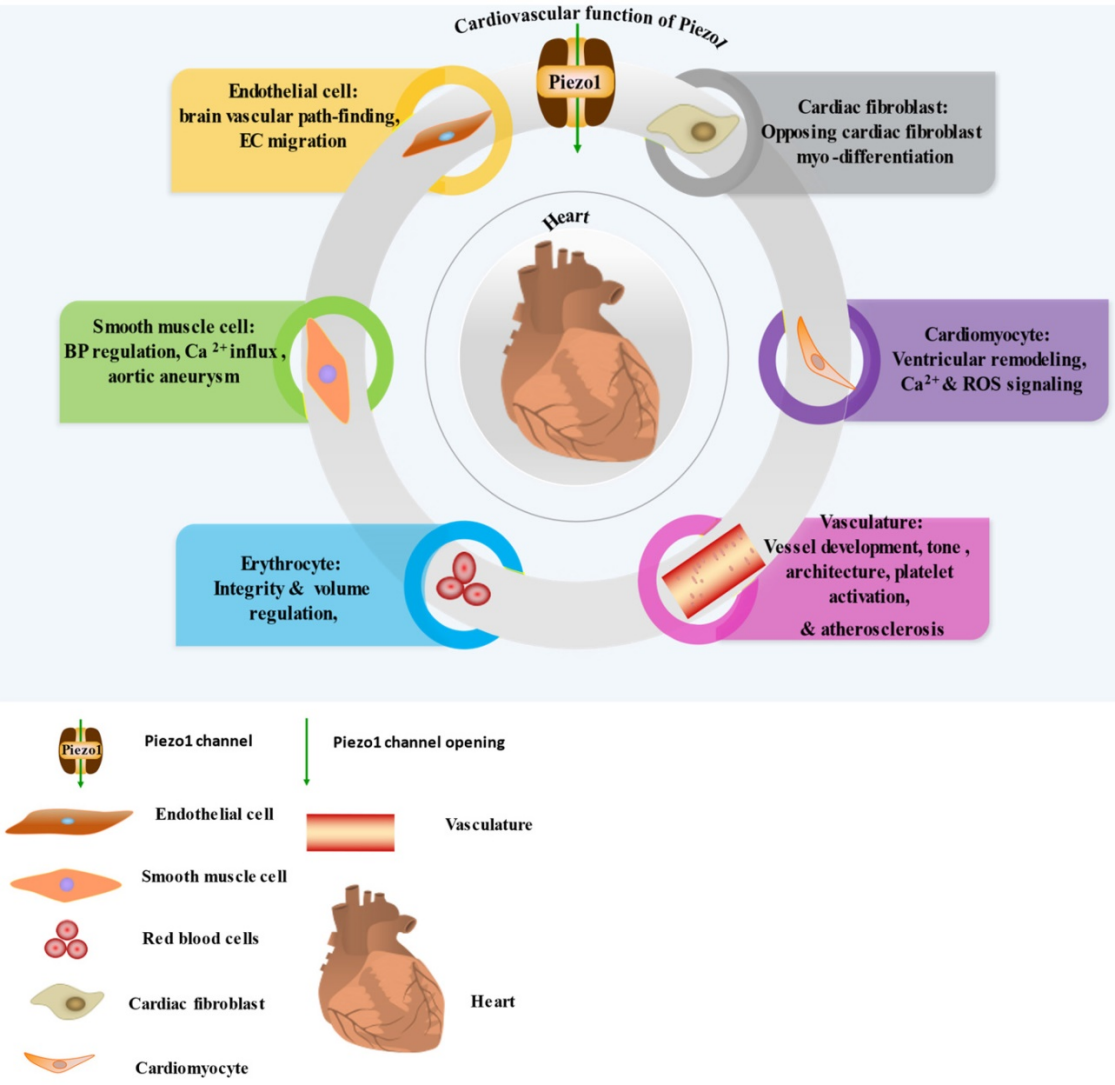
Cardiovascular expression and function of Piezo1, the detail Piezo1 role in each cell and tissue shown here explains in the main text of the review. In the diagram I-VI illustrating the significant functions of Piezo1 in EC, SMC, RBC, cardiomyocyte, cardiac fibroblast and vasculature.

**Figure 2:**
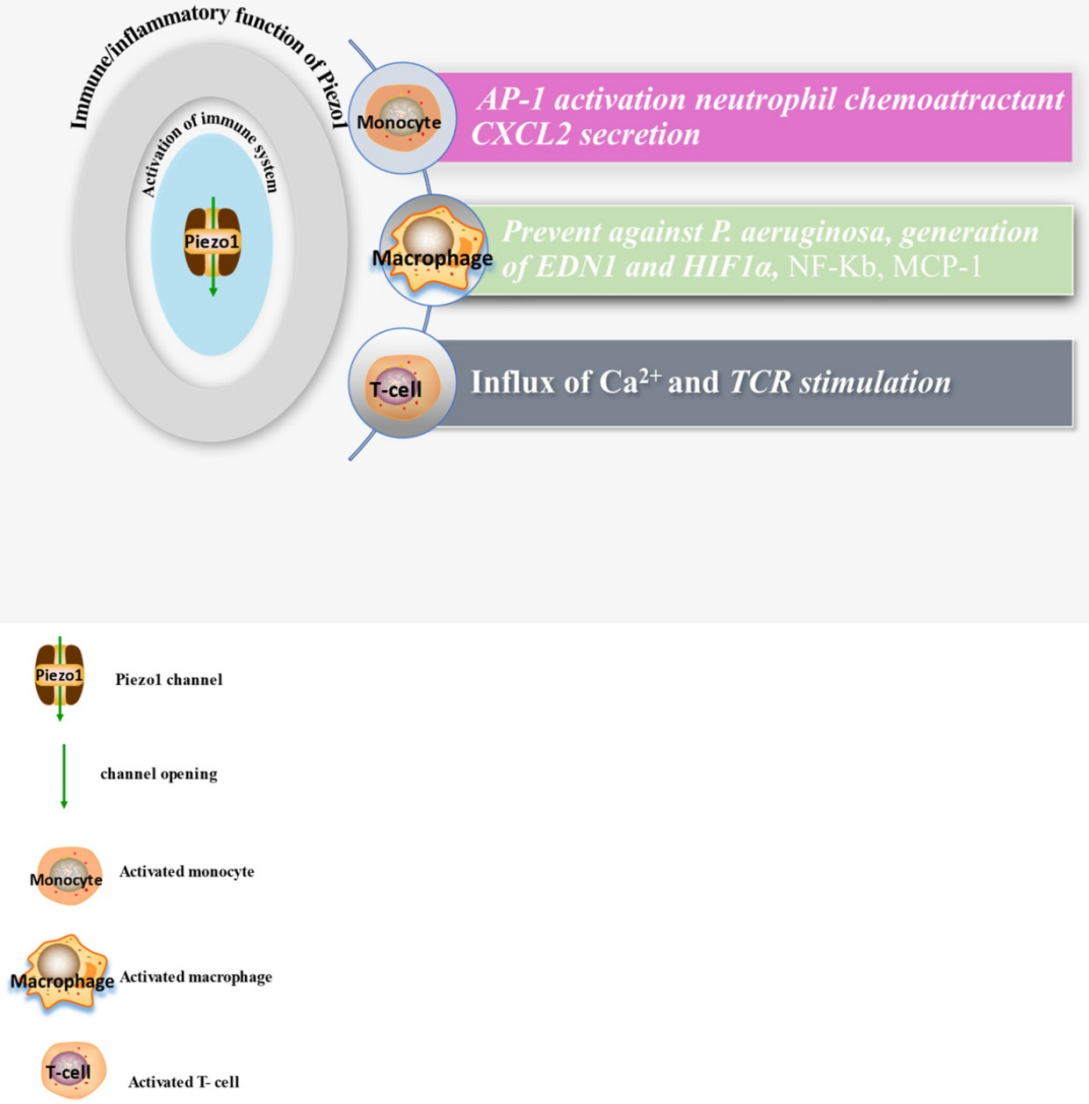
The diagram displays expressions and functions of Piezo1 in immune cells, comprising monocyte, macrophage and T-cells, illustrating the activities of these cell and secretion of chemokines after Piezo1 dependent activation as discussed in the main text of this work.

Piezo1 was reported to function in sensing and transducing of various mechanical forces, such as membrane tension, stretching 29 including shear stress 37, cyclical pressure 75 etc. To bring awareness of its validity as therapeutic targets, we highlight its various roles and mainly focus on cardiovascular including immune/inflammatory cells summarized in (Table 1) and illustrated in (Figures 1 & 2). Piezo1 is believed to have important roles in cardiovascular system. Increasing evidences revealed Piezo1 as cardinal player in the development, sustenance and normal function of the vasculature, inclusive of determining vessel architecture in embryonic and adult physiology. It also plays various roles in endothelium, including eNOS phosphorylation and vascular endothelial protein tyrosine phosphatase (VE-PTP) mediated endothelial dysfunction and hypertension in diabetes 76, contributing to the development and progression of atherosclerosis. Piezo1 is also player in cardiac fibroblast mechanotransduction, such as in fibrotic remodeling of heart, cell proliferation, differentiation of myofibroblasts, extracellular matrix turnover as well as paracrine signals 77. In mice, extensive interruption of the sprouting vessel and embryonic lethality was observed few days after heart begin to beats following its universal or endothelial specific disrupting 14, 78, 79. It is also necessary for outflow tracts as well as the formation of aortic valves 80. Piezo1 was also found to be significant in the regulation of lymphatic valve formation. Study by Nonomura and colleagues, using mice deficient of Piezo1 displayed decreased quantity of lymphatic valves, indicating the relevance of Piezo1 in lymphatic system 33. Another study in patient of familial lymphatic abnormality demonstrates the necessity of Piezo1 for normal functioning of lymphatic system. Compound heterozygous and homozygous mutants of Piezo1 were discovered in individuals with chronic lymphedema, ascites and pleural effusion related to inherited lymphatic dysplasia 24, 81. Piezo1 global knockout in mice leads to embryonic fatality 24, 38, 46, 82, 83, demonstrating the necessity of Piezo1 during embryogenesis. Piezo1 was also recently reported to functions in skeletal muscles regeneration by boosting the functioning of satellite cells 84. Additionally, severe defects in vessel maturation and remodeling, and disability in NO production and vessel dilation responding to flow was both observed following Piezo1 universal deletion or EC-specific disruption 37, 79. Piezo1 was reported to have versatile functions and it is responsible for detection and transduction of flow shear as discussed in Section 4, cell proliferation and migration in Section 5.3, immune cells activation and cytokine release in Section 5.2, in inflammation and atherosclerosis in Section 5.1 & 5.2. For review check ref. 14, 27, 40, 45, 85. Altogether these functions demonstrate Piezo1 potentiality as promising target for novel therapeutic innovation against atherosclerosis and other CVDs.

### 4.1 Piezo1 shear stress sensing

Shear stress resulting from blood flow is important phenomenon and vital for cardiovascular physiology. Piezo1 is well recognized and specialized shear stress sensor as proved by many evidences. Regardless of Piezo1 research infancy, the physiological and pathological relevance of Piezo1 in various aspects of cardiovascular was earlier reported 14, 86, 87. Piezo1 play significant roles in multiple aspects of cardiovascular including embryonic vessel development and regulating of brain vascular pathfinding discussed earlier, and its mutants were correlated with various disorders in human. It was reported that, embryonic vessel development which is believed to be evoked by shear stress was interrupted following the knockdown of Piezo1 14. In the study by Rode and colleagues, rapid activation of endogenous Piezo1 channel by shear stress was observed in excised membrane patch recordings from endothelial cell. Flow-induced depolarization of -43 mV resting membrane potential, recorded in membrane patches from endothelial fragment was found to be diminished and hyperpolarized, after conditional deletion of endothelial Piezo1 32, 62. However, shear stress phenomenon in *ex vivo* experiment was found to be restrained or absent after endothelial Piezo1 knockdown. These findings proved Piezo1 as bona fide shear sensor and it is required for transducing shear forces into biochemical signals. Despite its recognition as shear sensor and backed by bountiful evidences, we still have a fundamental question of what is the precise mechanism of Piezo1 shear sensing and what are the exact structures and domain involved in Piezo1 activation by shear stress, other mechanical and chemical stimulus? This is of course a critical and difficult question to address due to the technical limitations and infancy of Piezo research. Several studies disclosed lately, the mechanism and domains that may be involved in the modulation of channel activation and inactivation of the channel as discussed in Section 5.

### 4.2. Association of Piezo1 signaling with inflammation and atherosclerosis

**Figure 3:**
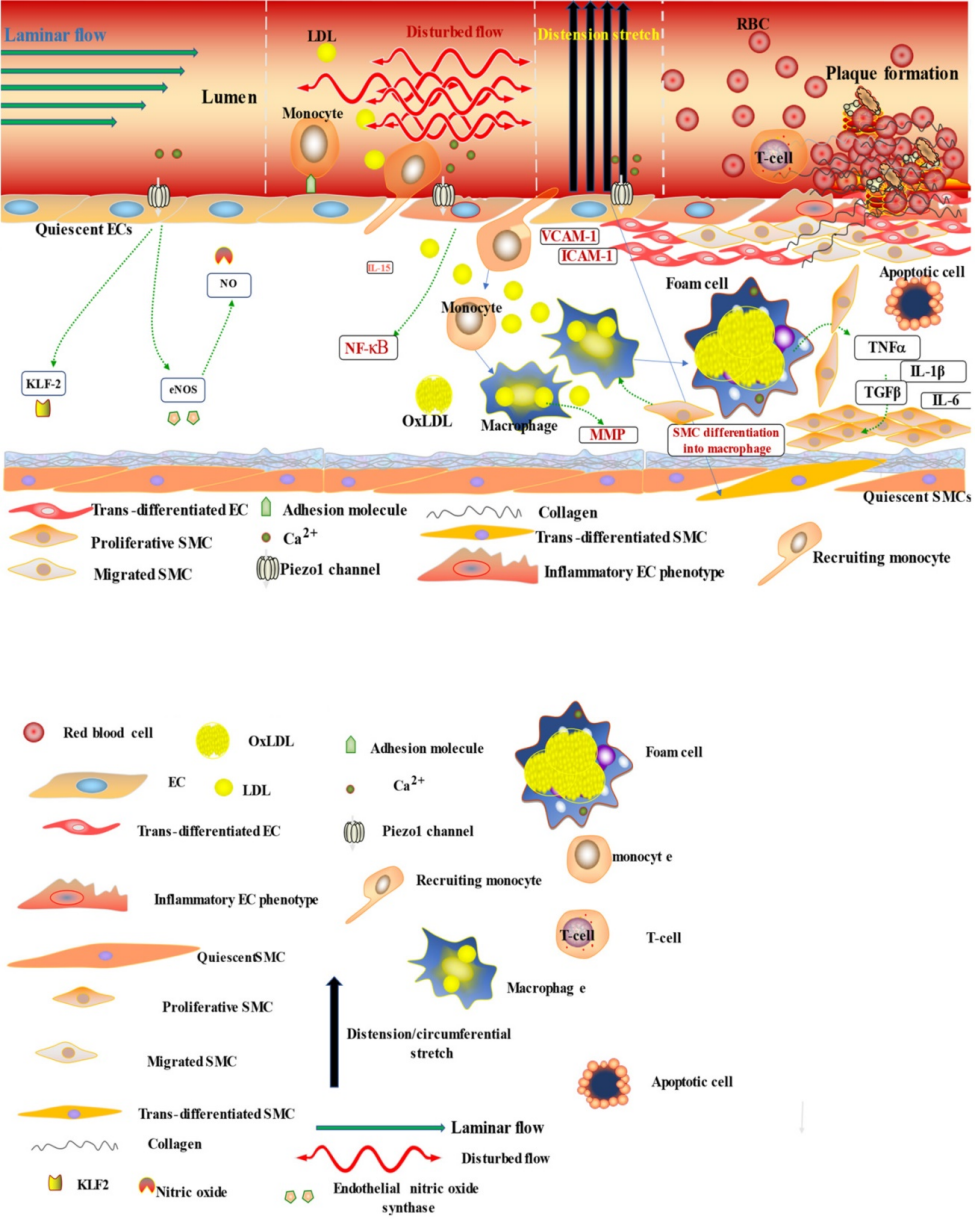
The diagram illustrates the atherogenic role of Piezo1 in different mechanical signals; first segment (vessel lumen) initiating Piezo1 dependent Ca^2+^ entry and trigger of atheroprotective signaling through EC activation and regulation of gene expression KLF2, NO and maintaining noninflammatory, non-proliferative/migrative quiescent EC. Second segment turbulent/oscillatory flow (red arrow) Piezo1 dependent triggering of proatherosclerotic signaling via EC activation to proinflammatory phenotype and immune/inflammatory cell recruitment, proinflammatory gene expression NF-κB, cytokine release (VCAM-1 ICAM-1) and result in cell proliferation and migration, leading to macrophage accumulation and form cell formation and atherosclerosis development. In addition to second segment, third segment distension/circumferential stretches (blue arrow) Piezo1 dependent stretch stimulation leading to trans-differentiation of EC to SMC phenotype, and SMC into macrophages, proliferation migration and apoptosis, release of growth factors TGFβ and cytokines TNFα, IL-6, IL1β, MCP-1 etc, contributing to disease progression and plague rupture. Fourth segment advanced atherosclerotic plaque and thrombus formation.

**Figure 4:**
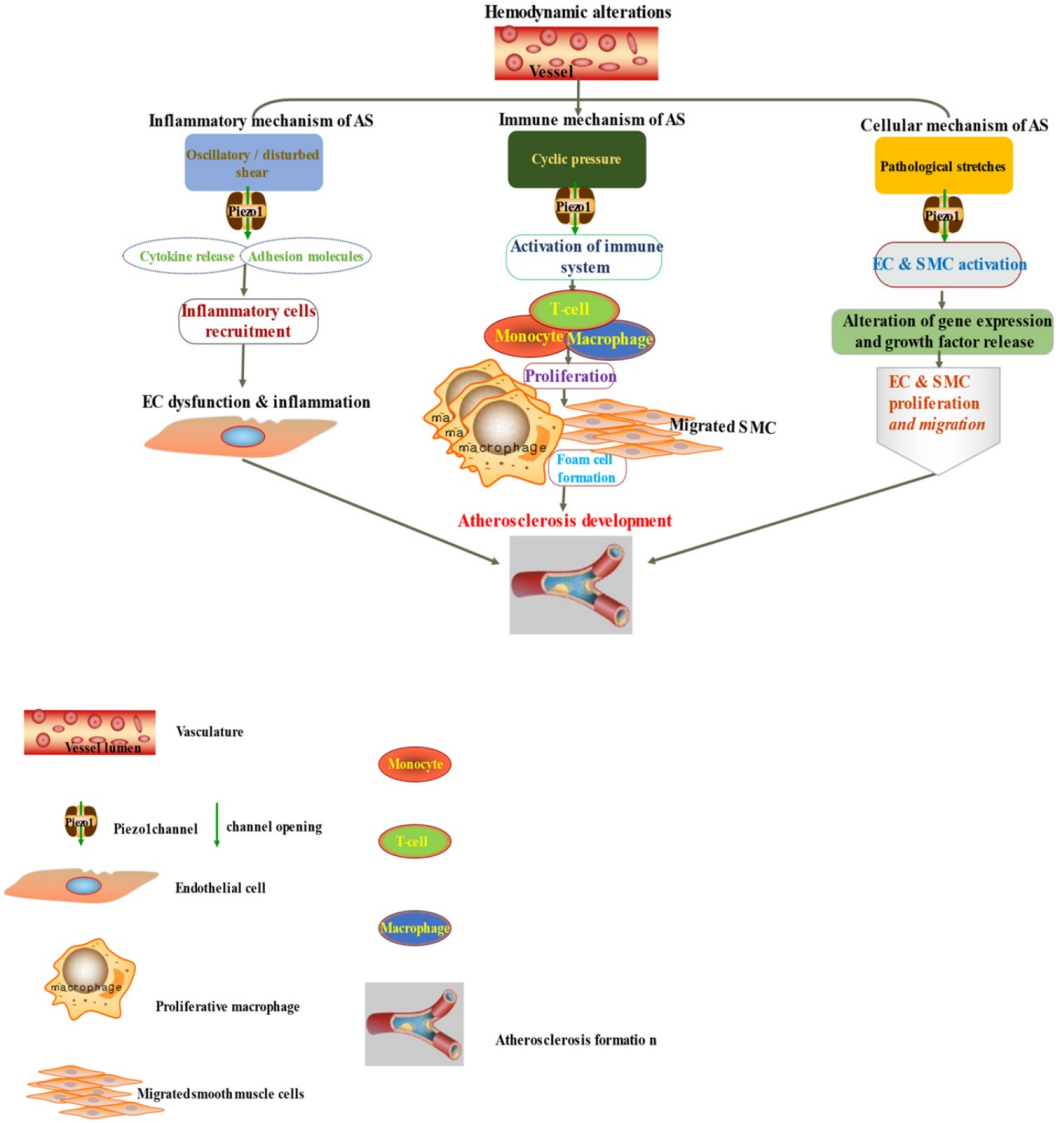
The diagram depicted Piezo1 dependent signaling; (left) inflammatory mechanism of AS by disturbed/oscillatory shear leading to the release of cytokines and adhesion molecule, inflammatory cell recruitment, EC dysfunction and inflammation, facilitating the development of AS, (middle) Piezo1 dependent activation of immune cells by cyclic pressure result in cytokine release, proliferation migration form cell formation and disease development, (left)Piezo1 dependent stretch activation of EC and SMC leading to changes in gene expression and growth factor secretion and result in proliferation and migration facilitating the development of atherosclerosis.

Atherosclerosis is a multifactorial and complex disease process of the vasculature involving various mechanisms and pathways in its pathogenesis. Accumulating evidences suggested the role of immune/inflammatory and other cellular process like cell proliferation, migration, apoptosis and remodeling in atherosclerotic pathogenesis 88-92 (Figures 3 & 4), and Piezo1 was reported to play crucial roles in aforementioned cellular activities. In this regard, it is considered pivotal for the integration of various pathways driving atherosclerosis pathogenesis and complications. Mechanosensitive Piezo1 channel play multiple roles in various aspects of cardiovascular including health and disease. Recent studies reported the involvement of Piezo1 in inflammation and immunity (discussed in Section 6.1), and cellular activities such as proliferation and migration (discussed in Section 6.2) and activation of proinflammatory mediators such as cytokine release, thus, coupling both traditional and other emerging risks to atherosclerosis.

### 4.3. Piezo1 mediated mechanical signals in inflammation and atherosclerosis

It is now appreciated that several inflammatory/immune mechanisms are involved in atherosclerosis. Inflammatory/immune cells such as monocytes and macrophages respond to mechanical stress via Piezo1 and display proinflammatory gene expression. Recent studies revealed the crucial role of Piezo1 in activation of T cell 93, NLRP3 inflammasome and assembling 94. Solis et al. unveiled the sensing ability of Piezo1 to cyclical pressure that triggers proinflammatory activities in immune/inflammatory cells (myeloid cells). In his effort to explore the effect of mechanical force on immune/inflammatory cells, he exposes immune/inflammatory cells to pressure cycle alteration *in vitro*, and found increased Piezo1-dependent cyclical hydrostatic pressure evokes proinflammatory gene expression in control, compare to Piezo1 disrupted cells. Furthermore, application of mechanical force to monocytes and macrophage initiates robust and specific pattern of Piezo1-dependent expression of proinflammatory and chemoattractive molecules *ex vivo*. Interestingly, absence of proinflammatory cytokines and chemokines expression was observed in the lack of Piezo1 75. On the other hand, shear forces encounter while extravasating demonstrated Piezo1-dependent upregulation of ICAM-1 in *ex vivo* model. In a study of flow model, endothelial Piezo1 or Gαq/Gα11 specific depletion in mouse, exhibit diminished inflammatory signals and activation of integrin with regression of atherosclerosis in athero-susceptible region 95. Conversely, in another study exploring the inflammatory role of Piezo1 in adipocytes, Piezo1 activation with its agonist Yoda1 show decreased TNF-α and MCP-1 expression, while blockade of Piezo1 with GsMTx4 increases TNFα, MCP-1, Il-1β, and Il6 expression 96. This suggest Piezo1 as potential hub in which distinct signaling pathways could diverge in responding to distinct mechanical cues and play a diverse role in different inflammatory mechanisms, in which it could trigger proinflammatory or anti-inflammatory and proatherogenic or atheroprotective signals depending on the type of stimuli (Figures 3 & 4), cell type and mechanism or pathway involved. Consistent with Solis findings, Piezo1 could encourage proinflammatory response that helps to get rid of harmful bacteria, while its deficiency could as well help prevent dysfunctional inflammatory injury in pulmonary fibrosis in mice. Additionally, myeloid cells Piezo1 depletion promoted endurance in polymicrobial sepsis and guard from cancer 97. It's worth noting that Piezo1 channel via Ca^2+^ signaling may regulate a number of vital cellular activities 98 including immune/inflammatory cells. Consistent with the concept that Ca^2+^ signals control important cellular functions like mitosis, differentiating and migration, expression and functioning of several endocrine secretion as well as transcription molecules, (Figure 3) that control immune/inflammatory cells homeostasis 97, 99-101. Therefore, Piezo1 channel can control inflammation and immunity via Ca^2+^ signaling as in pancreatitis, reported by Romac et al, in which Piezo1 agonist Yoda1 was applied and stimulated the influxes of Ca^2+^ that evokes calcium-dependent damage of the pancreas 102. As versatile mechanosensor, Piezo1 may play a role in various pathways and unexplored mechanisms, in addition to these already known. In addition to inflammatory/immune cells mechanisms, several experiments including flow models have been tested and demonstrated Piezo1 as player in inflammation, immunity and atherosclerosis 103, 104. Beside earlier reports including that of alberran Juarez and others, Zhang and colleagues also recently reported a unique Piezo1 mediated molecular process of inflammation and atherosclerosis via annexin A2 associated integrin triggering 105. Piezo1 abundant responses to OS was also shown to result in elevated Ca^2+^ entry leading to Gq/G11 induced integrin α5 activating and ligating into α5β1 through translocating into lipid raft, thereby encouraging FAK-dependent NF-κB triggering and the expressing of proinflammatory molecules VCAM-1 and ICAM-1 106 (Figure 3). Altogether, these findings support the idea that Piezo1 is not just limited to mediation of unidirectional or disturbed flow-evoked pro/anti-atherogenic signals, but also necessary for immune/inflammatory mechanisms of atherogenesis.

### 4.4. Piezo1 signaling in vascular remodeling

Remodeling is well recognized pathogenesis of CVDs which is triggered by various factors including variety of mechanical forces such as stretch, pressure, shear stress, cyclical stress that are fundamental in cardiovascular health and disease. SMCs and EC are major cells in the vessel, and they are continuously exposed to dynamic mechanical forces, which they sense via special mechanosensors such as Piezo1 and initiates regulatory responses. Mechanical force plays critical role in vessel remodeling, and mechanosensitive Piezo1 channel is well recognized sensor and transducer of various mechanical cues. Vascular remodeling comprises alteration in cellular proliferation, migration, apoptosis (discussed earlier) and ECM rearrangement including its synthetization and degrading 107, 108, hypertrophy, phenotypic switching, morphologic and functional alterations 109, the important mechanisms of atherosclerosis pathology and its progression. Study by Retailleau et al., precisely demonstrated earlier, the Piezo1 SMCs mechanosensing mechanism of vessel remodeling. The author reported that Piezo1 mediated the increase of cytosolic calcium as well as triggering of enzymatic activities (transglutaminase), necessary for arterial remodeling. He further ascertained that the depletion of smooth muscle Piezo1 in DOCA/salt/uninephrectomy hypertensive mice resulted in decreased media thickening without visible changes in endothelial layer 110.

#### 4.4.1. Piezo1 mediated mechanical signals in cell proliferation, migration, apoptosis

In cardiovascular, mechanical forces mediated by Piezo1, regulates various cellular activities like proliferation, migration, apoptosis. Occurrence of cellular proliferation, migration and apoptosis in macrophage, SMCs, and EC is crucial mechanism in the development of atherosclerosis plaque 111.

##### 4.4.1.1. Proliferation

*Smooth muscle cells.* Cellular proliferation is a fundamental process involved in vascular health and disease (Figures 3 & 4) 112-114, 115, 116. Among vascular cells SMC proliferation is crucial in atherogenesis, and cyclical stretching mediated by Piezo1 is commonly known to raise the SMC proliferation (Figures 3 & 4). Piezo1is crucial for sensing and transduction of mechanical cues, including shear stress 14, 34 and stretching 98, 110, 117-121, stretching was shown to work in synergy with OxLDL (oxidized lipoprotein) and norepinephrine in aggravating SMC proliferating by triggering of (ERK) signaling in both mice and rabbits 107, 122-125. Piezo1 is necessary for shear sensing 34, 126, 127, and shear stress was also known to speed up and regulate SMC proliferation via several mechanisms including MMP-2, MMP-9, PDGF, and the trigger of phosphatidylinositol 3 kinase (PI3K)-protein kinase B, ERK1/2, Akt signaling 128. Furthermore, upregulation of ERK and Akt trigger with sequential elevation of insulin-evoked cell proliferation was noted, following 15% stretch application to mice aortic SMCs 107, 128, 129.

*Endothelial cells.* In response to cyclical stretching known to be sensed and transduced by Piezo1 111, EC could proliferate in a mechanism that necessitates cell-cell interaction. Piezo1 was previously identified as significant player in angiogenesis, and EC proliferates, migrates, and coalesces in response to angiogenic stimulus, forming primitive vessel labyrinths that develop and remodels 108, 130. Additionally, excessive ECs proliferation was reported in supraphysiologic stretching, as seen from increased HUVEC expression of the oncogene c-Myc 108. This might significantly encourage vascular disorders including atherosclerosis, by inducing thickening of intima.

*Macrophages.* Macrophages also sense cyclic pressure through Piezo1 resulting in expression of several inflammatory mediators including of IL-1β 75, 131, IL-6, IL-8 as well as TNF-α 132 (Figures 3 & 4), which instigate inflammation as well as matrix metalloproteinases that stimulate cell proliferation 133 and induce plaque weakening 134-137. Additionally, macrophage activities can be control by Piezo1 through various mechanical cues, as it is highly expressed and functions in macrophage 75, considering matrix stiffness and topology were among mechanical factors that control the differentiating, functioning and proliferating of macrophages 132. Robbins reported that plaque macrophages accumulations and replenishing relies on resident macrophages proliferation instead of monocyte entrance 137, 138 (Figures 3 & 4), demonstrating and proving the significance of cell proliferation in atherogenesis, through Piezo1 mediated mechano-signaling.

##### 4.4.1.2. Migration

*Endothelial cell.* Studies reported the crucial role of Piezo1 mediated endothelial migrating toward VEGF, and eNOS triggering, result in NO production, crucial for cell migrating control amid vasculogenesis 27. It was also reported that, by responding to shear stress Piezo1 promote migrating and aligning in ECs. Recent study revealed the elevation in migrating speed in HEK293 cell line, noted as a result of overexpressing Piezo1, and there was nearly total inhibition of migrating after application of GsMTx4 or Aβ peptide 127. Therefore, these data suggest versatile migrative functions of Piezo1 with cell specificity in selective mechanism and signaling pathways.

*Smooth muscle cells.* Cellular migration is significant physiological process for development and tissue homeostasis or defense as well as in disease process. Mechanosensitive Piezo1 channel senses and transduces forces including cyclic pressure/stretches, shear stress, and membrane tensions which serves crucial function in cell migration. Both EC &VSMC experiences various forms of mechanical stresses, senses via Piezo1, arising from hemodynamic changes as in blood flow/pressure and circumferential stretches in (Figure 3), which results in aligning and phenotypic switching, leading to elevation in migrating and proliferating. In response to various mechanical stimuli VSMC migrates from media to intima as in atherogenesis (Figures 3 & 4) or healing, following vascular damage 107, 139, 140, and several mechanical stresses mediated by Piezo1 was reported to impacts cellular migrating through different mechanisms 141-144, for example in human VSMCs activation of migration via triggering of nuclear factor of triggered T-cells 5 nuclear translocating by 13% stretching for 24h, and increased migration through ERK dependent raised of myocardin expressing, by 20% stretching in mice VSMC 107. VSMC migration is well recognized mechanism of atherogenesis which induced by multiple factors including mechanical force which led to the migration of these cells from its normal location (media) to intima and proliferate as discussed above, contributing to plaque development and disease progression. Additionally, recent studies demonstrated the mechanical forces effects on both VSMC migration and phenotype alteration 145-147 (Figure 3) and release of various chemicals leading to pathological intimal thickening and plaque formation, the fundamental mechanism of atherogenesis.

*Macrophages.* In mechanosensitive tissues mechanical signal controls various macrophage functions including inflammatory responses 132. Immune cells, including macrophages, encounter various mechanical forces, such as shear stress mediated by Piezo1 in the circulation amid extravasation, and membrane deformation amid migration to the injury site. Beside migratory role of Piezo1 in various cell including stem cells 141, pancreatic satellite cells 143, and cancers 18, recent study reported that, Piezo1-dependent mechanical triggering of macrophages *ex vivo*, activates a robust and specific expression of pro-inflammatory as well as chemoattractive mediators 75, thus, initiating and promoting macrophage migratory responses to the inflammatory tissues.

##### 4.4.1.3. Apoptosis

*Endothelial cells.* Shear stress mediated by Piezo1 is recognized sensitizer of Tumor necrosis factor α (TNF-α)-related apoptosis-inducing ligand (TRAIL) in human umbilical vein endothelial cells (HUVECs) and cancer cells. Hope et al. reported the massive apoptosis elevation in number of cell lines (MDA-MB-231, COLO 205 and PC3 cells) after application of both Piezo1 agonist Yoda1 and Trail, which happen through Piezo1 dependent signaling via Ca^2+^ influxes which triggers calpain, while application of GsMTx-4 decreased shear induced TRAIL stimulation 148.

*Smooth muscle cells.* Mechanical force affects apoptosis and viability of vascular cells including ECs and VSMCs as they are exposes to different forms of mechanical stress of various intensity and magnitudes. Currently, Piezo1 is recognized as important player in mechanosignaling. Growing number of reports are increasingly proving the relevance of mechanical forces in cardiovascular apoptosis 149, 150. Apoptosis dysregulating is among the crucial mechanisms in CVD pathologies and raise in apoptosis was reported to be associated with atherosclerosis and heart failure. Piezo1 is essential for sensing and transduction of variety mechanical cues, and various mechanical forces are involved in the upregulation of VSMC apoptosis 107, 151, 152 (Figure 3). Study reported that application of stretching encourages cell death via the inducting of p53 upregulated apoptotic regulator in human VSMC with β1-integrin trigger of p38 MAPK in VSMCs of mice 107. Another study revealed that mechanical stressing evoked VSMC apoptosis rely mainly on p53 and mechanical stresses was shown to triggers p53 through Rac and p38MAPK mechanism 125.

*Other cell types.* Recent study reported that in alveoli, amid (ARDS) acute respiratory distress syndrome, stimulation of Piezo1 by mechanical stretching result in elevated intracellular Ca^2+^ evoked type II pneumocytes apoptosis, whereas, reduction in apoptosis was observed in the absence of Piezo1, which are in association with Bcl-2 pathway 153. Furthermore, another study of *ex vivo* model using shear, revealed Piezo1 mechanical force mediation of Ca^2+^ evoked Osteosarcoma cell apoptosis and inhibit proliferation. Additionally, another study *in vivo* reported growth encouragement of these cells after abrogation of Piezo1 gene expression in immunodeficient mouse 18, 154. Altogether, these results demonstrate the significance of Piezo1 in different apoptotic mechanisms and emphasizes its involvement in atherogenesis.

## 5. Piezo1 pharmacology

### 5.1 Piezo1 activators

Apart from mechanical forces, Piezo1 can chemically be gated. Recent studies, through high throughput screening technologies, small chemical compounds such as Yoda1 165, including the exploration of its Piezo1 activating mechanism and binding site 166, and Jedi1/2 74 was revealed (Table 3). Out of these compounds, Yoda1 is the most potent with maximum effective concentration (EC50) and bounding affinity to purified Piezo1 ranging from 10 to 50 µM. additionally, Yoda1 decreases the mechanical triggering threshold by balancing the gated conformations of the channel, which enables partial trigger of the channel without mechanical stimulus. Piezo1 was reported to have three possible interaction sites with Yoda1, 1 per subdomain. By characterizing chimeric hybrid channels combining Yoda1 sensitive and non-sensitive subdomains, the author demonstrated that, in one channel, only one sensitive subdomain is needed, and enough for Yoda1 evoked Piezo1 gating 53, 166 (Table 2). According to their finding, keeping a single pore of Piezo1 opened, the other subdomain stays gated. Furthermore, Piezo1&2 chimeric analysis show that the area with residues 1,961-2,063 is crucial for Yoda1 evoked Piezo1 trigger 166 (Tables 2 & 3), indicating the potency and strong affinity of Yoda1 to Piezo1, validating it as important tool in Piezo1 research. On the other hand, Jedi 1 & 2 are Piezo1 agonists with higher water solubility, prompt onset and rapid decaying than Yoda1. They, as well, initiate Piezo1 mechanical sensitivity and decelerate the inactivating of Piezo1 via extracellular side. For triggering and modulating Piezo1, Jedi acts on the site near the blade-beam activation thoroughfare distinctive from that triggered by Yoda1. Additionally, Piezo1 poking currents were synergistically evoked after coapplying of Yoda1 and Jedi 45, 74, which suggests Yoda1 and Jedi modulation of Piezo1 could be via distinctive mechanisms. Jedi 1 & 2 bind to 1-2,190 residues rather than CED, demonstrating possible Jedi 1 & 2 bounding sites as ELs of the propeller blades as shown by surface plasma binding assay. For Jedi evoked Piezo1 trigger the important mechanical transduction portions EL15-16 and EL19-20 of distal blades and L1342/L1345 of the beam proximity is required demonstrating that Yoda1 may be acting downstream of the beam whilst Jedi upstream of the blade (Tables 2 & 3). Studies demonstrated that various mutants abrogate different Piezo1 triggering force while others remain spared or other way round. Mutating of A2094W disrupted Yoda1 and Jedi1 sensitiveness while maintain that of membrane stretching. Similarly, mutants of beam L1342A/L1345A abrogates both mechanical triggering via cell poking and chemical stimulation of Yoda1 and Jedi1 while remain sensitive to stretching 74, 166. Mechanical trigger of stretching 167 and swelling was abolished by R2135A mutant without affecting Jedi2 and Yoda1 sensitiveness 166.

### 5.2 Piezo1 inhibitors

As new cation channel with versatile cardiovascular functions involving complex mechanisms and related pathway its pharmacology is still immature. Till date, GsMTx-4 168-171, gadolinium III (Gd3+) 18, 157 Ruthenium Red (RR) 29, 30, are the only recognized and widely used generic blockers of Piezo1, also used to inhibits several ion channels Table 3. GsMTx-4 is peptide toxin with reversible inhibition of Piezo1 activities and maximum Kd of 2 μM, commonly employed in Piezo1 researches. GsMTx4 modifies Piezo1 channel opening, its (L- and D-form) enantiomers blocks Piezo1, demonstrating its possible modulation of membrane tension rather than direct action on the channel itself. GsMTx-4 and RR have been employed in probing the Piezo1 triggering mechanisms as well as ion-conducting pore. RR If administered from extracellular side, inhibits the inward rather than outward current of mPiezo1/2 alone, suggesting its pore-inhibiting mechanism 45. Recent studies reported the recognition of other molecules, including Yoda1 analog named Dooku1 172 and the most recent compound Tubeimoside1 (TBMS1) extracted from ***Bolbostemma*** 173, both with reversible antagonizing capability of Yoda1-evoked Piezo1 trigger (Table 3). They were reported to exert their effect through selective binding site competition with Yoda1. Additionally, an amphipathic Abeta (Aβ) peptide was revealed to inhibit Piezo1 shear responses within the concentrations of fento to Pico molar. Aβ peptides are a membrane mechanic modulator with oligomer that was verified to have lesser potency. Nevertheless, by utilizing patch clamp, its inhibiting activity was not seen on Piezo1, using same concentration which blocked shear responses 127. However, the realization of these agents, both agonists and antagonists along with the latest advancements of Piezo1 topographic structure, could surely facilitates Piezo1 research for exploring and deep understanding of its structure-function-relationship in different conformational states and possible Piezo1 mechanisms in health and disease conditions.

### 5.3 Piezo1 channels drugability and therapeutic potentials

Aforementioned works including many not cited here, indicated the significance of Piezo1 in various pathophysiological processes, specifically cardiovascular. Due to the infancy of both Piezo1 and its pharmacology research, a lot is not yet known, but its functions are still emerging. The realization of Piezo1 and its pharmacology (Table 3) provides excellent opportunity, as it may be considered or used as indicator/biomarker for diagnostic, prognostic 174 and genealogical target for therapeutic approaches. Identifying Piezo1 as prognostic marker and low life expectancy related to its high expression in glioma patient 175 emphasizes the idea that Piezo1 could be attractive candidate for various clinical aspects of diseases including atherosclerosis in the near feature. Considering that Piezo1 is upregulated in some cells while downregulated in others, depending on cell type and mechanisms or pathway involved, the discovery of pharmacological agents (discussed above) for selective inhibition or activation of Piezo1 will be therapeutically inevitable. On the other hand, mechanomedicine/mechanotherapeutics 150, 176, emerged as new approach that demonstrate Piezo1 as doubtless and promising therapeutic target for cardiovascular diseases. As mechanical cues are among key determinants of cardiovascular homeostasis, sub-physiological and supraphysiologic mechanical stresses are implicated in abnormal proliferation, migration, apoptosis as well as immune/inflammatory processes and atherosclerosis, including other CVDs, and mechanomedicine could provide hopeful outlook for selective tuning of Piezo1 mechanotransduction for mechanostatic restoration and maintenance*.* Additionally, another therapeutic idea through Piezo1 is also promising approach, in which cellular responses can be mechanically regulated through modulation of the mechanosensory genes-circuit. Recent study disclosed an amazing novel immunotherapeutic innovation called chimeric antigen T- cell receptor (CAR-T cell), with efficacy against multiple soft tumors. For aimed and regulated deliverance, mechanosensitive CAR T cell was manipulated through regulating of mechanosensory capacity of Piezo1, in order that mechanical perturbation by ultrasound wave could trigger the CART T cells within selected area. It is a therapeutic approach with high geotemporal accuracy which could be employed for remote control stimulating of various cell species 177, 178, hence, CAR could also be employed for targeting immune/inflammatory and other related mechanisms of atherosclerosis. Altogether, these data demonstrate and validate the drugability and therapeutic potential of Piezo1 in cardiovascular, particularly atherosclerosis.

## 6. Perspectives

Piezo1 discovery, emergence of its roles in various aspects of cardiovascular and rapid development of its research demonstrated a promising potential of the field towards novel therapeutic innovation for clinical application targeting atherosclerosis and other CVDs. Regardless of the infancy of the field, recent advancement in the realization of Piezo1 topographical structure and the outstanding breakthrough of its pharmacological agents indicate Piezo1 as doubtless target for novel therapeutic approaches in cardiovascular. Despite lack of potent agonists and specificity of Piezo1 blockers, both agents are widely accepted and employed as tools in Piezo1 research. Among the challenges of Piezo1 research are; wide range of expression and functions in multiple cell types, limited knowledge of Piezo1 functions in association with other proteins, such as interplay with cytoskeletal structures 27, other membrane channels like Piezo2 as in Baroreceptor reflex 163, and TRPV4 182-184 etc. Nevertheless, in-depth understanding of the channels and related pathways overlapping, cross-talk as well as kinetic properties*,* cell specificity, and structure-function-relationship are required for elucidating how Piezo1 regulates particular disease process specifically atherosclerosis, to develop a high precision therapeutic approach through rational design of specific Piezo1 agonists and antagonists for obtaining effective therapies as well as avoiding or minimizing unwanted effects.

## 7. Conclusion

Due to relevance of mechanical forces in cardiovascular disease, mechanotransduction has attracts much attention. The role of Piezo1 mechanotransduction in vascular, immune/inflammatory cells, indicates that Piezo1 is involved in atherosclerosis, therefore Piezo1 is a promising candidate for therapeutic innovation against atherosclerosis and other CVDs. It may be engaged in number of unexplored pathophysiological mechanisms through different signaling. For example, Piezo1 is key transducer of mechanical cues into Ca^2+^ dependent signaling. Hence, through calcium, Piezo1 may contribute to atherosclerosis, though further study is required for this concept. Additionally, Piezo1 mechanical triggering leads to intracellular Ca^2+^ elevation and might consequently trigger the Akt/MTOR pathways through calmodulin (CaM)/CaM dependent protein kinases II (CaMII) which enhances cell proliferation and migration 18. Intriguingly EC was found to be trans-differentiated into SMCs with elevated markers of SMC including α-SMA, SM22-α, SM MHC, calponin as well as caldesmon1 upon exposure to mechanical stretching 108, 130, 185, 186, which is known to be sensed by Piezo1. This stretch-induced plasticity of EC could augment atherosclerosis progression, and might be Piezo1-dependent. This necessitates the additional effort to further explore Piezo1-induced-mechanical effects on vascular cells. Nevertheless, Piezo1 research has witnessed rapid development, for example recent advancement in high throughput technology such as atomic force microscope, patch clamp electrophysiology, cryo-electron microscopy including other computational biological tools and gene editing technology. We believe that through the utilization of these technologies Piezo1 could be studied further leading to insight into various CVDs and other diseases like cancer. Mechanomedicine/mechanotherapeutics using Piezo1 is among the promising strategies, for example in cardiovascular, selective tuning of Piezo1 to control the cellular vulnerability to excessive sub-physiologic or supraphysiologic mechanical forces, might block deleterious proliferation, migration, apoptosis or inflammation, to prevent the disease initiation or treatment through the reversal of disease pathogenesis. Piezo1 could also be employed for immunotherapy, especial using CAR for selective activation or modulating of several immune cells like monocyte/macrophage and T-cells either systemic or locally, to treat atherosclerosis and other CVDs, including COVID-19 and cancer as already reported for different cancer treatment strategies. Lastly, further elucidation of Piezo1 mechanisms regulating atherosclerosis should be the priority for future research as initial step for developing novel and effective therapeutic strategies for treating atherosclerosis.

## Figures and Tables

**Table 1:** Summarizing the cardiovascular and immune functions of Piezo1

Organ/Tissue	Functions	Associated disorder	Human/animal; mutation/genetic disruption, inhibition or Up-regulation/Downregulation	Cell type	Interactions	Downstream mechanisms	References
Heart	Ventricular remodeling	Heart failure	Human; upregulation	Cardiomyocytes	Hemodynamic/mechanical force, shear stress,	AngII dependent deterioration of failing heart	155
	Ca**^2+^** & ROS signaling	Cardiomyopathy	mKO	Cardiomyocytes	Hemodynamic/mechanical force, shear stress	Cardiac Ca**^2+^** homeostasis	156
	Opposing cardiac fibroblast myo-differentiating	NR	NE	Cardiac fibroblast	Pressure	IL-6 secreting	157
Blood/Lymph Vessels	Hypertension-dependent remodeling of artery, BP regulation, Ca^2+^ influx & ECM degradation in AAA	Arterial remodeling, Abdominal aortic aneurism	mcKO	SMCs	Hemodynamic force, shear stress, mechanical stretching	Rise in transglutaminase 2 activities	14, 27, 45, 110, 158
	Brain vascular pathfinding, vascular growth, vessel tone & remodeling, Control of placental blood flow, EC migration; Vessel architecture; Hyperpermeability of Pulmonary micro vessels	Atherosclerosis, Hypertension, Pericardial effusion	mcKO, Upregulation/dowmregulation	VECs	Hemodynamic force, shear stress, Cyclic pressure	Trigger of eNOS, AKT, KLF2, VEGFR2 & tyrosine phosphorylating, P2Y2 (purinergic receptor type 2Y2) receptor activation	14, 159
	Lymphatic valves development & sustenance	Generalized lymphatic dysplasia	mcKO (LFm humans)	LECs	Hemodynamic force, shear stress, cyclic stretch	actomyosin & VE-cadherin remodeling	14, 33, 45
	RBC Volume regulation, release of ATP	DHS and Plasmodium infection, Hemolytic anemia	GFm humans, cKd	RBC	Hemodynamic force, shear stress	Elevated cation permeability leading to dehydrating of erythrocyte	23, 160
	Shear-evoked influx of Ca2+, Platelet activation & thrombogenesis	NR	NE	Platelets	Shear stress	Not clear	45, 161, 162
	Baroreceptor reflex	Baroreflex dysfunctioning	mKO/mutant Piezo1&2	nodose & petrosa sensory cells	Hemodynamic force, shear stress,	Raise in mean arterial pressure	14, 163
Immune system	Prevent against P.aeruginosa, generation of EDN1 and HIF1α	Dysfunctional immunity against P. aeruginosa	mcKO/ inhibition	Monocyte/Macrophage	Cyclic pressure	Neutrophil recruiting and clearing of pathogens	75, 103, 104
	Influx of Ca^2+^ and TCR stimulation	Unknown	Downregulation/inhibition	T-cells	Mechanical force/ pressure	Calpain trigger, actin scaffold reorganizing, and stabilizing immunological synapse	93, 164

Abbreviations: TCR; T cell antigen receptor, GLD; generalized lymphatic dysplasia, ECM; extracellular matrix, mcKO; mice conditional knockout, NE; not explored, BP; blood pressure; DHS; dehydrated hereditary stomatocytosis; GFm, gain-of-function mutant, cKd; conditional knockdown, NR; not reported, AAA; Abdominal aortic aneurysm.

**Table 2:** Demonstrated activation and inactivation sites of Piezo1

Mechanical		Chemical	
Activation	Inactivation	Activation	Inactivation
**Sites/subdomain**			
N-terminal proximity 86 &300 sites 63 ELs of distal THU4 & THU5 45	External pore domain sites 2,422 & 2,425 63	EL15- 16, EL19-20 & L1342/L1345 at the beam 45, 74	L2475 & V2476 Residues 45, 74

**Table 3:** Summarization of current Piezo1 pharmacology

Agents		Binding site (domain)	References
***Activating agents***			
**Specific**	Yoda	C-terminal (ATM area)	53, 165
	Jedi1/2	L15-16/L19-20 area	45, 74, 179
***Modulating agents***			
		***Membrane lipid environmental alterations***	
**Unspecific**	Unsaturated-Docosahexaenoic acid	Slow inactivation	180
	Saturated-Margaric acid	Speedup inactivation	
***Inhibiting agents***			
**Unspecific**	GsMTx4	Pore of the channel	179, 181
	Ruthenium Red		
	gadolinium		
**Specific**	Tubeimoside1	Compete with Yoda1	179, 181
	Dooku1		

## Abbreviations

CVDs: Cardiovascular disease
VEC: Vascular endothelial cell
RBCs: Red Blood Cells
VEGF: Vascular Endothelial Growth Factor
PDGF: Platelet Derived Growth Factor
IL: Interleukin
MCP-1: Monocyte chemotactic protein-1
PECAM-1: Platelet Endothelial cell Adhesion Molecule
ICAM-1: Intercellular Adhesion Molecule
VCAM: Vascular Cell Adhesion Molecule
KLF: Kruppel Like Factor
NF-κB: Nuclear Factor Kappa B
eNOS: endothelial Nitric Oxide Synthase
ROX: Reactive Oxygen Species
HUVECs: Human umbilical vein endothelial cells
